# XXIV National Congress and XI International of the Spanish Society of Conservative & Aesthetic Dentistry: 102nd scientific meeting

**DOI:** 10.4317/jced.1122335667798

**Published:** 2022-11-28

XXIV National Congress and XI International of the Spanish Society of Conservative & Aesthetic Dentistry May 12-14, 2022 Pamplona, Spain Abstracts of the scientific communications follow.

