# Supplementary material for: XXIV National Congress and XI International of the Spanish Society of Conservative & Aesthetic Dentistry: 102nd scientific meeting
Source: J Clin Exp Dent. 2022 Nov 28;14(Suppl 1):S1–S15. doi: 10.4317/jced.1122335667798 (PMC9665179; doi:10.4317/jced.1122335667798)
Supplement: Related texts

# Abstract: Treatment of large apical lesions: About a case

**Authors:** P. Renauro Rius, R. Castillo Lafarga, D. Villanueva Ortiz, S. Gonzalez Bejarano, A. Pérez Cano, 0. Alonso Ezpeleta

## Introduction

Endodontic infections, even when root canal treatment is performed correctly, can continue to manifest different pathologies both clinically and radiographically.

## Case Report

A 36-year-old male patient comes to the clinic, totally asymptomatic for routine check-up. The radiographic examination shows an apical lesion in 1.1, 1.2 and 1.3. Clinically, sensitivity tests are negative in 1.1 and 1.2, and positive in 1.3. Emphasizing that the patient refers to having suffered a traumatism years ago, without being able to specify it. Endodontic treatment is started under magnification of teeth 1.1 and 1.2. When opening and during instrumentation, there is profuse and constant suppuration. It was decided to medicate intracanal (Ca(OH)2) during three visits. As there was no improvement, it was decided to change the intracanal medication to biantibiotic paste (Metronidazole and Ciprofloxacin), together with a curettage of the lesion through the fistula. After three weeks, when the symptoms and suppuration disappeared, it was decided to close the duct system. When performing the corresponding controls, the patient presented no symptoms and a reduction in the size of the lesion.

## Conclusions

The success of the treatment depends on the chemical and mechanical disinfection of the duct system. Therefore, knowing the existence of biofilm, how to deal with it and the treatment options available to us will help us to carry out a more accurate treatment with a better prognosis.

# Abstract: Intrusive dislocation with fracture of the buccal bone table

**Authors:** M. Calvo Ézara, J. Martin Cruces, M. Teulé Trull, P. Castelo Baz

## Introduction

The management of a trauma case is a demanding procedure for the professional due to the need for an immediate and effective action to know the possible treatment options, in order to properly choose the best plan for each case.

## Case Report

A 23-year-old female patient comes to the clinic 24h after suffering a trauma with lateral/intrusive dislocation of the 1.1. First of all, an extraoral examination is carried out followed by an intraoral examination. No damage to the perioral tissues or intraoral tissues was observed. A photographic record is taken to assess and compare the injury during the control appointments. Finally, a periapical radiograph is made where the obliterated periodontal space is observed. After performing a periapical radiograph, we determined that it was vitally important to perform a CBCT to obtain a more accurate view of the degree of dental intrusion, root position, assess the possible fracture of the bone table and angulation of the intruded tooth. The patient was wearing a fixed palatal orthodontic retention of the upper incisors during the trauma, which reduced the impact of the blow and facilitated subsequent splinting. The 1.1 was manually repo-sitioned and a 0.12 orthodontic NiTi unactivated wire was placed from 1.3 to 2.3 using brackets. After three months, the periapical radiograph showed the tooth to be stable, but it did not show any signs of vitality, so it was decided to perform endodontics. Finally, a direct composite reconstruction was made to replace the incisal edge and the fractured angle. Periodic controls were carried out after repositioning and endodontics to assess the evolution of the case.

## Conclusions

A correct diagnosis together with a multidisciplinary approach and long-term patient monitoring are essential factors for successful trauma management. 

# Abstract: Regenerative endodontic therapy in a dens evaginatus: a case report

**Authors:** R. Castillo Lafarga, P. Renauro Rius, L. Baroni Cañizares, L. Arméndariz Bandrés, S. González Bejarano, O. Alonso Ezpeleta

## Introduction

The interruption of root development in the young permanent necrotic teeth poses disadvantages for long-term survival rates, despite achieving biological closure of the tissues after a successful endodontic treatment. Procedures with calcium hydroxide require long treatment periods and seem to weaken the dentin structure. The use of mta shortens the time period, but they do not solve the lack of complete anatomical development. During the last decades, regenerative endodontic procedures have increased in popularity as they have the potential to resolve periapical pathology in addition to allowing the root finishing his development.

## Case Report

A regenerative endodontic procedure is performed on a maxillary central incisor with an anatomical anomaly in the cingulum known as dens evaginatus. The tooth fully develops the apex and remains asymptomatic for 5 years, when a acute apical periodontitis appears. During this time, the patient begins orthodontic treatment. After this new pathology, a novel root canal treatment is performed on the previously revitalized tooth.

## Conclusions

Regenerative endodontic therapies offer advantages over classical treatments designed for young permanent necrotic teeth. However, the new tissues generated are not histologically identical to the original ones and their clinical behavior can be unpredictable in some cases.

# Abstract: Guided endodontic. A solution for the management of calcified canals

**Authors:** E. Yáñez Pérez, M. Ruiz Piñón, P. Castelo Baz, M. Teulé Trull, L. Feijóo Artacho

## Introduction

Obliteration of the root canal or calcific metamorphosis occurs dangerously after traumatic injuries, espe-cially as a consequence of dental concussions and sub-luxations. This entity is characterized by a deposition of mineralized tissue inside the root canal and is often associated with a yellowish discoloration at the coronal level. This situation represents a great challenge for endo-dontic treatment, since it makes it difficult to locate, permeabilize and instrument the canal.

## Case Report

Patient who comes to the master’s degree referring spontaneous and intense pain in the central right incisor. The dental history records trauma to the same tooth four years ago. On examination, a change in coronal coloration and strong pain on percussion can be seen. On the periapical radiograph, calcification of the canal is appreciated. Therefore, CBCT is requested, where a periapical lesion is observed. After clinical and radiographic examination, pulpal necrosis and acute apical periodontitis were diagnosed. Given that in the CBCT the lumen of the canal begins to be appreciated in the apical third, it is decided to carry out root canal treatment using guided endodontics, followed by internal bleaching. A 3D scan is performed to design, together with the CBCT, a guided splint; thus planning the path of insertion and depth that the drills must reach. With the splint in the mouth, the drills are introduced to the planned length and the canal is negotiated and permeabilized with a preK file. The conformation of the root canal is performed with Endogal rotary instruments and it is filled with the continuous wave technique. The treatment is concluded with internal bleaching with sodium perborate.

## Conclusions

Guided endodontics is a safe, conservative and predictable technique for the treatment of calcified anterior teeth up to the mid-apical third.

# Abstract: Autotransplantation of 4.8 with immature apex instead of 4.6

**Authors:** M.P. Villarte Diestre, B. Lainez Rubio, O. Valencia de Pablo, R. Peñuelas Calvo, S. Poc Sola, O. Alonso Ezpeleta

## Introduction

The advantages provided by dental autotransplantation and its high percentage of clinical success make it a therapeutic option to consider to replace non-maintainable teeth, being the ideal moment for its realization when 2/3 to 3/4 of the donor tooth root has been formed.

## Case Report

A 16-year-old woman is referred for the presence of a previously treated 4.6 with asymptomatic apical periodontitis. After performing the root retreatment, the lesion healed, but after 2 years, the image worsened again and we observed an apicomarginal defect with loss of vestibular cortical bone. Due to the lack of success of the previous treatments, it was decided to extract 4.6 and auto-transplant 4.8 with an open apex in the position of the first molar. With the help of CBCT, we plan the case and make a replica of the donor tooth. We carried out the extraction of 4.6 and the socket was prepared until a correct settlement of the replica was achieved. Next, we proceeded to the atraumatic extraction of 4.8 and it was positioned in the receptor socket in less than 10 seconds. Previously, we had adapted the gingival margins in the recipient socket by suturing and stabilized the tooth with braided-wire splinting, which we removed after two weeks. After one year of follow-up, the patient is asymptomatic, vitality is positive and both root development and the formation of new bone around it can be observed.

## Conclusions

Autotransplantation is a predictable technique as long as we follow a strict clinical protocol and an adequate selection of the case. In donor teeth with immature apex, it can allow root development and maintenance of pulpal vitality. The incorporation of replicas of the donor tooth is crucial to minimize extraoral time.

# Abstract: Autotransplantation of two immature third molars. Case report

**Authors:** A. Zemhoute Tifaoui, V. Faus Matoses, I. Faus Matoses, C. Ruiz Sánchez, V.J. Faus Llácer

## Introduction

The main objective of dentistry is to preserve dental structures for functional and aesthetic purposes, from pathologies such as caries or periodontal disease and other factors as dentoalveolar trauma, cause tooth loss and this forces the dentist to resort to techniques to replace these spaces with fixed, removable prostheses or resort to implants. All of these options have potential disadvantages. However, tooth autotransplantation is considered as a therapeutic option when replacing a lost tooth and has received considerable attention worldwide due to its high level of clinical success, especially in children and adolescents, although it has also been used in adults with good results. This surgical technique consists of transplanting a tooth from one site into another in the same individual, involving the transfer of impacted or erupted teeth to extraction sites or prepared surgical sites. This case report describes a successful case of a 12 years-old patient with no medical history of interest, treated with two autotransplantation of open-apex third molars (No. 1.8 and 3.8) in place of the hopeless first molars (No. 1.6 and 3.6) that were extracted due to non-restorable coronal destruction. Clinical and radiographic follow up of 2 years exhibited complete bone regeneration, asymptomatic vital pulp and continuous growth of the root of the transplanted molars. Tooth autotransplantation is an interesting treatment option in case of tooth loss in growing patients, since dental implants are contraindicated until complete growth. 

# Abstract: Dens invaginatus type TITb in a lateral lower incisor; an anatomical challenge

**Authors:** A. Vicente Galicia, E. Labrador Ortiga, A. González Dominguez, S. Poc Sola, A. Villanueva Ortiz, O. Alonso Ezpeleta

## Introduction

Dens invaginatus is a developmental anomalytesulting in a deepening or invagination of the enamel organ into the dental papilla prior to calcification of the dental tissues. The reported prevalence is between 0.3-10% and its exact actiology is unclear yet.

## Case Report

A 16 years old female patient who comes to the clinic for a general revision. Radiographic examination showed a type TIb dens in dente (confirmed by CBCT) in tooth 3.2, which presented asymptomatic apical periodontitis. At this time it is decided to treat with an endodontic treatment. With the help of a guided splint designed with a computer-guided implant planning software, the access cavity was more exactly and therefore less invasively. Between two visits, calcium hydroxide was used as an intracanal medication to get better chemical disinfection. After testing an apical diameter of 55 in dens in dente, it was decided to perform apexification with bioceramic materials, however, the other canal was filled with the “continuous heat wave” technique. Follow-up appointments at 3, 6, 9, 18 months, it accomplished all the necessary clinical and radiographic tests. Finally, 3 and a half years later, it was observed an absence of clinical signs and a radiographic reduction of the periapical lesion.

## Conclusions

The therapeutic planning of a dens in dente type Tllb will depend, apart from a good diagnosis, on the use and interaction of the new technologies that we have within our reach in all dental arcas.

# Abstract: Autotransplantation of a third molar: Therapeutic alternative for the failure of a lower molar

**Authors:** J. Dominguez Cachón, P. Castelo Baz, M. Ruiz Piñón, J. Bahillo Varela, A. Arnau Vidal

## Introduction

In recent years, autotransplantation of third molars has become a very useful tool that can be carried out in an apparently simple way, in the event of the loss of a molar. Carrying out this treatment provides an increase in aesthetics, function and complete bone development of the socket, in relation to other types of treatments that are perhaps more commonl y accepted, with a similar medium-short-term prognosis.

## Case Report

The present case is a 39-year-old patient who attends the Master’s Degree in Endodontics at USC derived from the Master’s Degree in Periodontics at the same university, in order to do, together, the treatment plan of a 4.7 with the presence of lesion apical and persistent symptomatology for several weeks. The pertinent diagnostic tests are performed and a diagnosis of vertical fracture in the mesial root is established. lt was decided to propose to the patient an autotransplantation of tooth 2.8 in the socket of 4.7, endodontic treatment and subsequent restoration with a lithium disilicate overlay. At 6 months, the patient is reviewed and a complete recovery of the function of the piece and an optimal state of the hard and soft tissues are observed.

## Conclusions

After carrying out this clinical case and with the endorsement of the existing scientific literature, we can conclude that the autotransplantation of a mature third molar in a post-extraction socket is a predictable treatment with a high success rate, provided that the planning is carried out correctly and following all the clinical steps precisely.

# Abstract: Management of immature permanent teeth after dental trauma

**Authors:** B. Laínez Rubio, E. Labrador Ortiga, A. Vicente Galicia, S. Poc Sola, S. Abizanda Guillén, A. González Domínguez, O. Alonso Ezpeleta

## Introduction

Nowadays, endodontic management of the necrotic im-mature permanent tooth remains a challenge, as root development is disrupted, resulting in thin dentine walls prone to fracture, an unfavourable coronoradicular ratio, and wide apices that do not make it a good candidate for conventional root canal treatment.

## Case Report

An 8-year-old patient presented at the clinic after suffering a dental traumatism. Clinical and radiographic diagnostic tests were carried out. Two months after the trauma, fistula and necrosis were observed in tooth 2.2. Endodontic regeneration of tooth 2.2 was performed in two sessions, using 2 as intra-canal medication, with the aim of increasing the root length and solving the existing apical periodontitis. At the 12-month check-up, necrosis of tooth 2.1 was observed, which was treated by an apical plug of Biodentine®. After a 30-month followed up, healthy periapical structures and apposition of mineralised tissue on the root of tooth 2.2. were observed.

## Conclusions

It is important to know the management of dental trauma and the sequelae that can result from it. We will favour regenerative endodontics in immature teeth with divergent apices, thin dentine walls and short roots, while apical plug will be our gold standard in teeth with an adequate coronoradicular ratio and open apex. Both treatment options show high survival rates. 

# Abstract: Multidisciplinary management of dental trauma: a case report

**Authors:** B. Laínez Rubio, A. Villanueva Ortiz, D. Villanueva Ortiz, P. Villarte Diestre, A. González Dominguez, O. Alonso Ezpeleta

## Introduction

Dental trauma is considered an emer-gency and must be treated immediately in the dental office, crown fractures and luxations of permanent teeth are the most commonly occurring of all dental imjuries.

## Case Report

This case describes a dental trauma in a 27 years old man during a sports accident. On clinical and radiographic examination, using CBCT, it is observed that the anterior sectors present the concurrence of various traumatic injuries: 1.1 complicated crown fracture with lateral luxation and fracture of the alveolar socket wall; 1.2 and 4.1 complicated crown fracture with subluxation; 1.3 complicated crown-root fracture; 2.1 and 4.2 uncomplicated crown fracture with subluxation; 3.1 enamel infraction with subluxation. In an emergency appointment after the trauma, the palatal fragment of 1.1 and the crown of 1.3 were extracted, the vestibular bone table of 1.1 was repositioned and the upper arch of 1.4 to 2.3 was splinted (attaching the crown of 1.3 for aesthetic reasons), and the lower arch from 3.3 to 4.3. The therapeutic approach combined the treatment of tooth canals with pulp exposure, the extraction and immediate implantation of a non-restorable tooth, and the rehabilitation of the affected teeth using posts and layered composite veneers.

## Conclusions

A multidisciplinary approach in the treatment of dental trauma leads to the best possible results with respect to the rehabilitation of the function and aesthetics in the affected sector.

# Abstract: Clinical protocol of internal bleaching

**Authors:** P. Mendez Hernandez, R. Mendez Diaz, J.J. Gaite Garcia, M. Teule Trull, J. Martin Crosses

## Introduction

Internal bleaching has an approximate clinical success rate of 60%, but 90% of the patients were satisfied. The etiology can be: during the necrosis process, post-traumatic pulpal hemorrhage, endodontic errors, restorative materials or external pigmentation. Absolute contraindications for its performance are: periapical lesion, incorrect endodontic treatment or periodontal disease. Before doing it, we must correct these clinical circumstances. Our material of choice is the use of chemically pure sodium perborate.

## Case Report

We will illustrate with several clinical cases, the protocol that we recommend for performing internal bleaching, as well as various tips for greater clinical success.

## Conclusions

Internal bleaching, performed with chemically pure sodium perborate, is a safe and predictable treatment, both as a final treatment and as an auxiliary, prior to prosthetic rehabilitation of the tooth.

# Abstract: Rehabilitation of a patient affected by tetracyclines and attrition using new ceramics

**Authors:** A. Sánchez Redondo, V. Faus Matoses, I. Faus Matoses, C. Ruiz Sánchez, V.J. Faus Llácer

## Introduction

Tetracyclines constitute a group of antibiotics, covering a broad spectrum in their antimicrobial activity. Among its side effects are discoloration in the temporary and permanent dentition in different degrees. One of the objectives of restorative and conservative dentistry is to resolve these clinical cases by combi-ning functionality and aesthetically. Based on the fundamentals of minimal invasive dentistry, new materials, techniques and technology have emerged. Among these new materials we find the Celtra Duo porcelain that is presented in blocks for CAD/CAM processing and contains lithium silicate reinforced with zirconium dioxide that provides great resistance to bending. Thanks to its glass content, it visibly improves opalescence and fluorescence. In addition, the opacity changes according to the number of crystallizations that are carried out, thus achieving optimal aesthetic results. There are different alternatives to the treatment of severe tetracyclines such as aggressive preparations and intentional endodontics that are avoided thanks to these new ceramics which ensures that the color does not show through.

## Case Report

A 48 year old female patient decided to undertake dental care concerning her teeth color caused by tetracicline usage. Clinical analysis manifested esthetic problems including severe tetracycline discoloration and attrition. The treatment plan consisted of a complete oral rehabilitation through the placement of 22 partial coverage indirect restorations made with Celtra Duo porcelain by CAD/CAM system. Due to the minimal invasive preparation, it was possible to maintain pulpal vitality and complete adhesion to enamel.

## Conclusions

Thanks to the minimal invasive conservative and restorative management, satisfactory results were obtained at an aesthetic level since it was possible to completely mask the discoloration caused by tetracyclines.

# Abstract: Yellow spots: From the micro to macroabrasion. A case report

**Authors:** P. Freire Nieto, J.G. Martínez Colmenares, J. Roldán Cubero, L. Jané Noblom, M. Roig Cayón

## Introduction

Intrinsic dental discolorations are described as pathological processes that affect the color of the tooth in its structural components, either the enamel, the dentin-pulp complex or both.

## Case Report

A 15-year-old male patient attends the Restorative and Aesthetic Dentistry Department of the Universitat Internacional de Catalunya (UIC) in order to improve both functionally and aesthetically the upper anterior sector. On clinical examination, intrinsic yellow, oval-shaped and well-defined discolorations are observed in 1.1 and 1.2, of unknown etiology. In addition, at cusps 1.3 and 2.3, another type of white discoloration is seen, compatible with enamel hypoplasia. As a treatment for the removal of dental stains, a microabrasion protocol was followed with the product OpalustreTM (Ultradent), a water-soluble paste with 6.6% hydrochloric acid and silicon carbide microparticles. A small amount of paste was applied with a rubber cup (OpalCup) at low revolutions (500rpm) for 60 seconds, and a maximum of 5 applications. The use of fluoride or potassium nitrate was recommended to avoid postoperative sensitivity. After re-evaluation, the yellow spots in 1.1 and 2.1 persisted, although with less intensity, and therefore macroabrasion was carried out with rotary instrumentation. To do this, a diamond ball bur was used at high revolutions (200,000 rpm) and with refrigeration, deepening the enamel until the stains disappeared. As a restorative material, the injectable fluid composite technique (Anaxdent) was used thanks to a transparent silicone key obtained from the printed model of the patient’s diagnostic wax-up.

## Conclusions

The removal of stains on the dental surface can be achieved by combining various conservative techniques, such as the application of chemical-abrasive products, without having to use other prosthetic procedures such as adhesive ceramic restorations. 

# Abstract: Prosthetic rehabilitation of a conoid tooth

**Authors:** R. Méndez Díaz, P. Méndez Hernández, J.J. Gaite García, M. Teulé Trull, J. Martín Cruces

## Introduction

We can define conoid teeth as those teeth in which the mediodistal width of the incisal edge is much smaller than in the cervical. It is an anomaly in the shape of the teeth, with the affected tooth having a conical appearance. Conoid teeth have a prevalence, about 1%. We can perform various types of treatments to improve their aesthetics and function: from direct composite restorations, porcelain veneers, or full coverage crowns.

## Case Report

A 15-year-old male patient attends the Restorative and Aesthetic Dentistry Department of the Universitat a fully covered translucent zirconium crown is made (because it is the best material for this type of preparation) with practically no dental preparation, in order to preserve the greatest amount of material. The only thing we were looking for was to remove the aprismatic layer of enamel, an insertion axis for the placement of the crown, for which a very slight knife-edge preparation was carried out. A digital impression and crown were made in a prosthetic laboratory. The fundamental thing was to give an aesthetic and functional aspect to our tooth, inserting it in the smile line.

## Conclusions

The new materials, the new technologies, make a substantial improvement of our aesthetic treatments.

# Abstract: Biologically Oriented Preparation Technique (B.O.P.T.): Case report

**Authors:** B. Saz León, J. Martín Cruces, M. Teulé Trull, P. Castelo Baz, M. Calvo Ézara

## Introduction

The biologically oriented preparation technique is a simplified prosthodontic technique that consists of two different clinical phases: the deep prepa-ration of the tooth without a finish line, and the shaping of the new gingiva based on the adjustment of the provisional crown. This technique is intended to limit the clinical com-plications associated with typical fixed prosthesis restorations, based on predisposing factors such as: - Inadequate quality and quantity of keratinized gingiva - Iatrogenic effects during treatment (trauma by clamps, threads, carving...) - Chronic inflammation due to prosthetic errors - Traumatic brushing

## Case Report

Three clinical cases are presented in which the biologically oriented preparation technique will be performed on upper central incisors, in which the aesthetic demand was the main reason for carrying out said technique. Associated with aesthetics, we find other limitations of previous restorations such as gingival recession, marginal dyschromia, chronic inflammation, and marginal contour asymmetries. In conventional preparations for fixed prostheses, we find different possible designs for the finishing line. In contrast, the biologically oriented technique stands out for a vertical preparation with no finishing line. At first, this technique may seem more invasive at the soft tissue level. But the truth is that thanks to the fact that the emergence profile is eliminated with rotary diamond instruments in the cementoenamel junction to create a new prosthetic union, it will be perfectly adapted to the gingival margin.

## Conclusions

This procedure is intended to achieve a change in the shape and thickness of the soft tissues surrounding the tooth, creating a new anatomical crown with a prosthetic emergence profile that simulates the shape of the natural tooth. This will allow for better integration of the restoration and better stability of the tissues around the tooth. 

# Abstract: Complete oral restoration on tetracycline affected teeth using ceramics without teeth preparation

**Authors:** T. Moradian, V. Faus Matoses, C. Ruiz Sanchez, I. Faus Matoses, A. Zubizarreta Macho, V.J. Faus Llacer

## Introduction

Tetracycline is a strong antibiotic which can be incorporated into skeletal and dental tissues causing permanent staining and is influenced by dosage, length of treatment and the stage of tooth mineralization. Treatment options can be bleaching, veneers or crowns. No preparation ceramic veneers are an established treatment in clinical practice for preserving enamel and it has a superior bonding potential due to its adhesion to enamel.

## Case Report

A 50 years old male patient, pursued dental care complaining about discolorations caused by tetracycline usage and spaces between his teeth. Clinical examination revealed type II malocclusion division I, positive Bolton analysis, tetracycline discolorations and excessive gingival display in the maxillary anteriors. The treatment began after presenting him with a mock-up and his treatment options. Later, a periodontal phase was carried out, in which the goal was to increase clinical crown height in the maxillary anteriors by gingivectomy which rectified the gummy smile. Ultimately, a restorative phase was carried out, with placement of porcelain feldspathic veneers without teeth preparation and a full ceramic bridge which was fabricated covering the second premolar and molar in the fourth quadrant. Generally, obtaining sufficient space for the ceramic is carried out by tooth preparation, thickening the vestibular ceramic, increasing vertical dimension or a combination of these factors. Due to dental position and size (positive Bolton and negative torques) in this case, we achieved sufficient vestibular space to accommodate the thickness of the ceramics, avoiding fracture and covering the color of the substrate. Simultaneously, increased the vertical dimension, obtaining occlusal space for the ceramic and reducing overbite.

## Conclusions

Veneers are effective for covering tetracycline dis-colored teeth and no preparation veneers are easier to adapt and execute in cases with sufficient space, it also has a long term shade stability on the veneer and tooth interface.

# Abstract: Orthodontic-conservative timing in the treatment of agenesis of lateral incisors

**Authors:** M.T. Pagés Corral, V. Faus Matoses, I. Faus Matoses, V.J. Faus Llácer, A. Zubizarreta Macho, C. Ruiz Sánchez

## Introduction

The “intra-orthodontic mock up” is an innovative concept which is used as a guide for a minimally invasive restorative treatment, without a dental preparation or with minimal invasiveness. With the proposed approach, the intra-orthodontic mock-up will be the link in the interdisciplinary treatment, being placed in an intermediate phase within the treatment. This is how “intra-orthodontic mock-up” will guide the orthodontist to achieve an ideal tooth position, which will have been approved with the restorative from both an aesthetic and functional point of view. The aim of this case report is to demostrate a minimally invasive ortho-restorative management for the dental wear and missing upper lateral incisors with the help of an intra-orthodontic mock-up.

## Case Report

This Case report involves a 34-year-old patient with aesthetic and functional problems. Clinical analysis showed class 2 on both sides, a slight deviation of the midline, gingival smile and crowding. Moreover agenesis of upper lateral incisors and wear on the posterior teeth. The treatment plan included a first restorative phase which includes the restoration of posterior teeth wear. After that orthodontic phase was carried out to correct the dental position and malocclusion and to achieve a correct meshing of the teeth. After that the intra-orthodontic mock up is placed, to achieve the correct dental proportions in the maxillary dentition. The second phase of orthodontics was carried out.

## Conclusions

Finally, after the completion of Orthodontic therapy and removal of the “intra-orthodontic mock-up”, sufficient space will have been obtained to place the final restorations without the need for tooth preparation.

# Abstract: Clinical protocol of an internal whitening of a traumatized tooth, in a medically compromised patient

**Authors:** M. Anache D´Abate, P. Méndez Hernández, R. Méndez Díaz, J.J. Gaite García, M. Teulé Trull, J. Martín Cruces

## Introduction

The literature tells us that we should not perform in-ternal bleaching on a traumatized tooth. Our clinical experience tells us that only incorrect endodontic, advanced periodontal disease or periapical pathology make a tooth ineligible for whitening. To do this, we must have a strict protocol, and follow a series of steps that make the treatment feasible and reliable.

## Case Report

We present a case of internal whitening of a patient with a long-standing trauma, a medically compromised patient. All these antecedents make this treatment not the choice. A strict protocol, dental treatment, which consists of an apical plugging with EvoMTA, apicectomy, adequate coronal sealing to perform whitening and all this carried out under the experience of a research group with hundreds of cases treated, make this treatment predictable. in your result.

## Conclusions

Internal bleaching, performed with che-mically pure sodium perborate, is a safe and predictable treatment.

# Abstract: External cervical resorption of a lower premolar: Treatment with surgical Access

**Authors:** L. Feijóo Artacho, R. Miguens Vila, P. Castelo Baz, J.J. Gaite García, I. Varela Aneiros

## Introduction

Pathological root resorption consists of the loss of hard dental root tissue as a result of unwanted odontoclastic action due to an alteration in the stability of the periodontal ligament, this is not a frequent pathology. It can be classified based on the height of the lesion, its circumferential extension, and the proximity to the root canal. The treatment and prognosis of said injury will depend on its location and extension.

## Case Report

Patient with no medical history of interest comes with pain in tooth 3.5 with an evolution of 1 month. After performing the diagnostic tests, the existence of root resorption was suspected, so a cone beam computed tomography was requested. In said test, the presumptive diagnosis was confirmed. The present external cervical resorption extends to the coronal third of the root and apically to the crest, with a circumferential extension of less than 90º and with pulp involvement; Therefore, after performing the root canal treatment, it was decided to perform a surgical approach with coronary lengthening. The lesion was filled with composite, preserving the space necessary for the bone crest, and performing an exhaustive polishing, to achieve a correct insertion of the connective tissue in the biological width, ensuring good periodontal health. Follow-up is carried out at 6 months.

## Conclusions

External cervical resorption is not a frequent pathology; however, an early diagnosis and adequate treatment are a key factor for a good long-term prognosis. To carry out a correct treatment, it is important that the clinician has skills and knowledge in both the periodontal and endodontic-restorative fields.

# Abstract: Multiple apical fracture and multidisciplinaty treatmente: about a case

**Authors:** I. Amezqueta Fernández, J.J. Gaite García, A. Garrido Aguirre, K. Herguedas Manso, A. Laloma Gómez, M.J. Ortega Chillón

## Introduction

We present a case with a multiple apical fracture in an upper canine that, after its non-surgical conservative treatment, has allowed us to avoid the usual approach of extraction as the first standard choice, for the subsequent orthodontic treatment with aligners.

## Case Report

Patient who comes for orthodontic evaluation, with the particularity of presenting signs of occlusal trauma, mobility and pain on percussion and palpation in the apical area of 1.3, with positive vitality. The orthopantomography shows a horizontal root fracture of the apical third of 1.3. For orthodontic treatment, we are faced with the dilemma of choosing between extracting the fractured canine (which would be the usual first choice in a case like this) or trying to preserve it. We opt for the conservative option, by performing root canal treatment and we show the results of the evolution of the tooth problem.

## Conclusions

We reflect the importance of Conser-vative Dentistry in multidisciplinary treatments and how a tooth with a poor prognosis has withstood the use of aligners after conservative treatment.

# Abstract: Invasive external cervical resorption: endodontic, surgical and restorative approach

**Authors:** B. Magallanes Camón, L. Usieto Gonzalez, L. Armendáriz Bandrés, S. Abizanda Guillén, D. Villanueva Ortiz, O. Alonso Ezpeleta

## Introduction

External cervical resorption is a pathology that affects dental structure, compromising pulpal health and tooth survival. The resorbed tooth structure is replaced by highly vascularized tissue, which can become visible through the residual enamel, giving the affected tooth a pinkish hue known as a “pink spot.” They are classified based on their extension and location with respect to the root of the tooth. It has been related to trauma, orthodontics and more variables, although its etiology remains unclear.

## Case Report

Patient who comes to consultation because a “pin-kish-colored spot” is observed in 2.1. On clinical examination, he presented discomfort to percussion, gingival inflammation and increased sensitivity. After this exploration and after performing the pertinent radiographic tests (complemented with CBCT), the diagnosis of external cervical resorption type II of Heithersay and 1Bp of Patel was established. There are multiple treatment options, and for this particular case, the endodontic and restorative approach was decided upon, performing debridement through surgical access to the cervical area of the tooth and subsequently restoring the lost tooth structure with nanohybrid composite. Both phases were performed in a single appointment. In (the) follow-up at 18 months, the complete healing of the case is observed.

## Conclusions

Due to its different clinical manifestations, often asymp-tomatic, the correct diagnosis and treatment planning is crucial to guarantee optimal results, both functional and aesthetic. 

# Abstract: Orthograde Retreatment: upper molar with post, perforation, separated instrument and apicoformation

**Authors:** L. Usieto González, B. Magallanes Camón, S. Abizanda Guillén, A. Pérez Cano, R. Peñuelas Calvo, O. Alonso Ezpeleta

## Introduction

Orthograde retreatment offers very favorable long-term results, which together with CBCT allows to achieve high success rates.

## Case Report

A 48-year-old female patient is referred by her gene-ral dentist with discomfort in tooth 1.6 with previous endodontic treatment over 15 years ago. In the clinical examination, we observed that tooth 1.6 had a zirconium crown and did not present a fistula or suppuration and the probing was slightly increased in the distobuccal area. The vertical and lateral percussion tests were positive and the palpation tests were negative. Radiological examination revealed a radiolucent image in the apical area of tooth 1.6, and in order to obtain a more precise diagnosis, a CBCT was performed. The retreatment of the canals is carried out, which will consist of the elimination of a carbon fiber post and the separated element, as well as the treatment of the mesiopalatine canal not previously treated and the sealing of a perforation present in the palatal root together with the apex formation of said root. In the control three years after treatment, the patient is asymptomatic and radiographically a very important decrease in the radiolucent area is observed.

## Conclusions

With CBCT we can plan treatments and make more precise decisions, which together with the magnification, allows us to address complex cases as well as accidents during endodontic treatment. 

# Abstract: Comparison of different angles in reciprocating motion for reduction the cyclic fatigue

**Authors:** N. Mariño Fernández, M. Teulé Trull, P. Castelo Baz

## Introduction

To analyze the variables that can influence the resistance to fracture due to cyclic fatigue of the instruments, specifically the most suitable angles of rotation to which our motor must be programmed during the reciprocating movement. Morover, determine which is the influence of the mass and taper of the instruments.

## Material and Methods

The total sample size for the study was of 80 rotary instruments divided in 4 groups of 20 rotary instruments (15.03, 20.04, 25.04, 25.06) of the Endogal system cach. They were used in the Motopex endodontic motor. Each group is divided into 2 subgroups: The first one consists of 10 instruments actioned in reciprocating motion at 400rpm with the angles of 150°/30°; the second, consists of 10 instruments actioned in reciprocating motion at 400rpm with the angles of 120%/30°. All instruments were operated in a stainless steel artificial canal simulator with a 90° angle of curvature and a diameter of 1.4mm. Time to fracture of Each instrument was determined.

## Results

The instruments 15.03 and 25.06 of the Endogal® system reciprocating at 120°/30° were more resistant to cyclic fatigue, even doubling the time to fracture compared to alternating motion 150°/30°, while instruments 20.04 and 25.04 did not present differences in the time until the fracture occurred between both groups.

## Conclusions

According to the results obtained in this study, the reciprocating movement at 120/30° is safer than the reciprocating movement at 150º/30° when we used the 15.03 and 25.06 Endogal® instruments. There were no notable differences in the rest of the instruments in the different angles used.

# Abstract: Calcium ion reléase on four silicate-based cements

**Authors:** R. Herrera-Trinidad, V. Vera Rodríguez, B. Hernando Dumaraog, P. Molinero Mourelle, M.L. Mena Fernández, V. Vera González

## Introduction

The aim of this research is to evaluate the calcium ion release.

## Material and Methods

Four bioactive materials are tested (ProClinic MTA; Angelus MTA; ProRoot MTA; Biodentine). Last three millimetres of the palatal canal root of acrylic upper molars are filled with each cement. After, they are set in a phosphate buffered saline at 37ºC and 99% of relative humidity. Measures are made by atomic adsorption spectroscopy (AAS) at three hours; 24 hours (one day); 72 hours (three days) and at 168 hours (seven days).

## Results

Significate differences are observed in pair comparisons for ion calcium release at 3 hours, 24 hours and 72 hours.

## Conclusions

All analysed groups have an ion calcium release during the first seven days (168 hours). There are significate differences among the analysed groups at 3 hours, 24 hours and 72 hours. It can be determined that, in a sevenday period, silicate-based cements that have the most ion calcium release are Biodentine™ and ProRoot MTA.

# Abstract: Effectiveness of irrigation systems in curved conduits. Bibliographical review

**Authors:** B. Martínez Martín, P. Castelo Baz, M. Teule Trull, R. Miguens Vila, J.J. Gaite Garcia

## Introduction

To evaluate the difficulty of irrigation in teeth with curved ducts and the effectiveness of current irrigation systems for cleaning and disinfection.

## Material and Methods

We looked for in vitro studies that compared the efficacy of positive pressure irrigation (PPI), passive ultrasonic irrigation (PUI), continuous ultrasonic irrigation (CUI) and a new activated irrigation system, continuous apical negative ultrasound (CANUI), in teeth extracted with straight and curved ducts. The electronic platform PubMed was used for this search between November and January. The words “Endodontics”, “irrigation”, “activation systems” and “root channel” were used. Articles were searched in English and Spanish over a period of 10 years.

## Results

In straight roots, the results showed a significantly greater penetration of the irrigant in lateral ducts when the CUI system was used compared with PUI and PPI. However, there were no differences between CUI and PUI when it came to main ducts. On the other hand, the CANUI system showed a considerably greater penetration of the irrigant than that of the PUI system, both in main and lateral straight ducts and in curved ones at working length. Finally, PPI obtained the lowest penetration of the irrigant compared to the CUI, PUI and CANUI systems, both in straight roots, in main and lateral ducts, and in curved roots.

## Conclusions

Irrigation is a fundamental part of the chemical, biolo-gical and physical debridement of the root canal system. The difficulty of penetration of the irrigant in curved ducts has not yet been completely solved with current systems. CANUI, improves penetration in the lateral ducts and even the working length in straight roots and curves, however, more similar studies are needed for its comparison. 

# Abstract: New mechanical instrumentation systems: Systematic review

**Authors:** P. Sánchez Llobet, C. Rico, J. Mena

## Introduction

Comparing, in a descriptive way, by reviewing the available scientific literature, the systems of rotary instrumentation in endodontics considering parameters such as: surface treatment, cutting section and flexibility and duct conicity and deformation in the face of cleaning. In addition to assessing the cleaning of the dentinal barrel, formation of the duct, fracture of the instrument and fracture as a function of number and time of use.

## Material and Methods

A systematic review was conducted in the MEDLINE (via PubMed), Wiley Online Library databases, bet-ween December 2020 and April 2021. In addition, a manual search was performed to identify relevant studies regarding the selected articles. A ten-year search was conducted and articles in any language other than English or Spanish were excluded, as were those evaluating rotational systems other than those studied. A meta-analysis was not possible due to the heterogeneity of the studies.

## Results

A total of forty-two articles met the inclusion criteria. All studies compare the rotating files studied. Vente-seven studies compare the resistance to cyclic fatigue, obtaining a more favorable result in Reciproc, Profile, Profile Vortex, Hyflex CM, Hyflex EDM, NeoNiTi, Genius Files and Mtwo; eleven the ability to center and maintain dental anatomy, where Twisted File turned out to be the most conservative file; four the surface treatment of the new mechanical instrumentation systems being, M-Wire and CM-Wire the ones that confer the greatest results.

## Conclusions

The evidence suggests that the use of mechanical instrumentation systems is a good daily clinical practice, however, more information is required, with studies that provide greater homogeneity regarding the three main properties included in this review, emphasizing the unification of a standard methodological design with respect to the property evaluation method in question.

# Abstract: Cyclic fatigue resistance of 25.06 files in continuous rotation

**Authors:** U. Esmorís Suárez, R. Piñeiro Turrado, A.B. Dablanca Blanco

## Introduction

The aim of this study was to compare the cyclic fatigue resistance of three different systems 25.06 files in continuous rotation.

## Material and Methods

A sample of 30 files of 25.06 from three different systems: D from Endogal (N=10), X2 from Protaper Next (N=10) and NeoNiti A1 from Neolix (N=10); were submitted to a cyclic fatigue resistance test. They were introduced inside an artificial stainless steel canal with a 90º angle of curvature and 5 mm radius. The instruments were rotated in continuous motion at 350 rpm and torque 4, until their fracture. Time until fracture and the length of the fractured instrument segment were recorded to evaluate the correct positioning of the instrument inside the canal. Statistical analysis of the data was done by a mean difference.

## Results

The Endogal instruments were the ones that lasted the longest time until fracture, the difference was statistically significant compared to Protaper and Neolix. No statistically significant differences were found regarding the length of the fractured segment.

## Conclusions

Within the present study limitations, instrument D from Endogal had significantly higher cyclic fatigue resistance than X2 from Protaper Next and NeoNiti A1 from Neolix.

# Abstract: Study of the mechanical strength of different types of fluid resin composites

**Authors:** A. Burches Feliciano, M. Monterde Hernández, L. Ilzarbe Ripoll, M. Segovia Soriano, C. Pérez Roig, K. Mora

## Introduction

The objective of this study was to calculate the mechanical strength of various flowable composites, (the injectable composite resin technique) with observation of the pressures exerted to check at which fracture occurs. The pressures are quantified and descriptive and graphical statistics are performed in order to verify if there is any statistical correlation.

## Material and Methods

The composites to be used in this study were: Tetric EvoFlow (Ivoclar), Renamel microhybrid (Cosmedent), Renamel microfill (Cosmedent), Grandioso heavy flow (Voco). Previously, a diagnostic wax-up was made on the study model. On this wax-up, the injection splint of addition silicone (elite glass). Subsequently, with the silicone placed on the model, it was placed in a pressure cooker for 5 minutes. Small openings were then made in the silicone matrix, perforating with a drill of each tooth. An applicator was used to check that the syringe of flowable resin entered and exited unobstructed. In the models without the wax-up, etching was done with 37% phosphoric acid for 20 seconds, it was removed by washing with abundant water. The surface was dried for the application of 3M universal adhesive, on the surface, the excess was removed with air and light cured with a polymerization lamp. After checking the insertion it´s correct, the flowable resin was injected through the perforations. These steps were performed on 20 samples distributed in groups of 5 models for each of the flowable resins. Once the described models were manufactured, the mechanical resistance of each one of them was measured with a testing machine. Finally, the results were evaluated with statistics (both descriptive and to look for correlation).

## Conclusions

The project is still in progress. Awaiting results and statistical analysis.

# Abstract: Optical and visual properties of two materials in two mínimum thickness

**Authors:** F. Ruocco

## Introduction

Today, the demand for aesthetic restorations resembling perfect natural teeth is very high. There are many factors that then determine the optical properties of a restoration. For this reason it is important to study the optical properties of the restorations at different thicknesses using a spectrophotometer, but the visual part which is of first impact at the sight of a smile is no less important. Compare the translucency and opalescence parameter of the two types of restorations with the two different thicknesses (0.4/0.8 mm) using a spectrophotometer (Vita Easyshade). In addition, the restorations will also be compared visually (diffused light) to understand if there are differences to simple sight.

## Material and Methods

8 blocks will be considered (4 lithium disilicate y 4 nano-hybrid composites): 4 IPS e.max CAD (2 LT A2 y 2 LT A3) y 4 Grandio blocs nano-hybrid composite CAD (2 LT A2 y 2 LT A3) with 2 different thicknesses (0.4 mm and 0.8 mm). All samples will be analyzed with a spectrophotometer. Subsequently 3 blocks will be considered (2 identical and 1 different to turn, up to the use of both materials in different thicknesses and different shades) and will be visually analyzed for 10 seconds by 8 people first with diffused light. The data will then be statistically analyzed using the variance accounting for (VAF) coefficient with Cauchy - Schwarz inequality and one-way ANOVA.

## Conclusions

The process is still ongoing. Waiting for results and statistical analysis.

# Abstract: Comparative study of radiopacity in current restorative materials with digital radiography

**Authors:** B. Hoyas Cuenca, A. Victoria Escandell, M.J. Segovia Soriano, M. Monterde Hernández, A. Pallarés Sabater, R. Hoyas Aguilar

## Introduction

The objective of this study has been to evaluate the radiopacity of the different materials currently used to cement and seal the remaining dental structure.

## Material and Methods

Eight materials were prepared, in the form of a disc, six blocks of CAD/CAM resin and two resinous cements, with thicknesses of 0.5, 1’0, 1.5 and 2 millimetres, comparing them with the enamel and dentin of a dental sample prepared in the same thickness as the samples. An aluminum wedge was manufactured to be able to compare the samples with it according to ISO 4049 and ISO 9917. The samples of material, dental and aluminum wedge were placed on a photostimulable phosphorus plaque and exposed to x-rays, to be able to visualise the greyscale of the materials by digital image. The statistical analysis was carried out with the Mann-Whitney tests.

## Results

We have found that for all materials, in their different thicknesses, radiopacity is lower than enamel and dentin. Pending more data, we can observe for the moment, that Voco Bifix cement and the Grandio Voco A2 LT block are the ones that have most resembled the radiopacity of dentin and enamel in all thicknesses, moreover Lava Ultimate of 3M and Brava FGM in different thicknesses having the most statistical differences compared to dentin and. Compared to the aluminum wedge, we observed greater similarity in Grandio de Voco A2 HT with the thickness of 2 millimetres. In the rest of the thicknesses we do not obtain enough evidence to differentiate radiopacity.

## Conclusions

Voco Bifix cement and the Grandio Voco A2 LT block are the ones that have most resembled the radiopacity of enamel and dentin in their thicknesses. 

# Abstract: Resistance comparision between Graphene and hybrid as post-endodontic restoration material in conventional crowns and endocrowns

**Authors:** M. Oliver Padilla, M. Monterde Hernández, K.D. Mora, I. Cascales Caballero, L.M. Ilzarbe Ripoll

## Introduction

The aim of this study is determining the resistance to axial force between graphene-reinforced resin and ceramic-reinforced resin comparing conventional crown restorations and endocrown-type inlays.

## Material and Methods

In the present study two restorative materials with two different types of milling for post-endodontic restoration are compared. As group 1 we use graphene (G-CAM, Graphenano Dental) and as subgroups we have two types of carving: 1.1 carving for endocrown and 1.2 carving for conventional crown. As group 2, we use ceramic-reinforced resin (Lava Ultimate, 3M ESPE) with the same subgroups as the previous group. Sample obtention was carried out by selecting two lower molars from the UCV database, carved for endocrown and crown, respectively. Later, both lower molars were printed in liquid resin doped with graphene for 3D printing. As a sample, 40 teeth have been printed in total (20 of each material and subsequently, 10 of each carving). The pulp chamber of the resin teeth with carving for a conventional crown is filled with composite (Ceram.x Spectra ST DENTSPLY SIRONA). The crowns were designed and printed by CAD/CAM (CEREC) and were cemented by an adhesive system. Later, the crowns were subjected to axial force until fractured.

## Conclusions

The present study is still ongoing; therefore, we await the results and conclusions to be obtained. 

# Abstract: Tooth preparation for veneers with guided splints versus silicone key

**Authors:** A. Grigoryan

## Introduction

Digital advances have enabled dentistry to plan accurately the teeth preparation in fixed prosthesis treatments, in addition to the prospect of performing minimally invasive aesthetic treatments by controlling the exact amount of dental tissue removed. Objectives The objective of this study is to compare the marginal and internal fit of cemented veneers made using two different guided preparation techniques: on the one hand, through the FirstFit® system using digitally guided splints and, on the other hand, teeth preparation guided by silicone keys taken from a printed digital wax-up.

## Material and Methods

The study will be carried out in 2 geller models. It will be prepared from canine to canine in both models, in one with the FirstFit® system and in the other one with the conventional technique using silicone keys. The veneers will then be cemented on these models and each tooth on the model will be cut sagittally with the veneer cemented, then scanned and the scans superimposed to measure discrepancies. On the other hand, the cut made in each tooth of the two geller models will be observed, comparing the marginal and internal adjustment using the surgical microscope.

## Results

Waiting for the results.

## Conclusions

Waiting for the results.

# Abstract: Comparative study of the fit, microleakage and fracture of provisional crowns designed by CAD/CAM

**Authors:** O. D’Arrigo Vinci

## Introduction

To evaluate marginal fit, microleakage and fracture of provisional crowns in the anterior sector manufactured with 3D printing technology, CAD/CAM milling technology, direct conventional technique and graphene therapy.

## Material and Methods

A total of 30 provisional crowns were constructed and divided into four groups according to the manufacturing methods: Group 1: direct conventional technique; Group 2: computer-aided design / three-dimensional (3D) computer-aided manufacturing technology Group 3: 3D printing technology. An STL file was used for each of the groups. Where the marginal fit of each crown cemented with TEMP BOND NE was studied using a digital electron microscope. All samples were previously immersed for 24 hours in methylene blue. Subsequently, an analysis was carried out prior to the marginal adjustment, observing the microleakage. Then each cemented crown was subjected to compressive tests until its fracture, in a ZwickRoell Z100 universal testing machine; each sample was observed under a digital electronic microscope for its final analysis. All results were tabulated.

## Conclusions

It is in a state of analysis with the statistician. 

# Abstract: Fracture resistance on maxillary centrals with minimal ferrule restored with three different techniques

**Authors:** W. Strauss

## Introduction

Anterior teeth with endodontic treatment and loss of dental structure are usually restored with fiberglass posts or metal posts, and are susceptible to failures that can cause tooth loss due to fracture. There are new techniques such as endocrowns that could be an alternative in current dentistry. Objectives To evaluate the fracture resistance of the transverse compressive forces of the restorations and root fracture, with different restorative techniques.

## Material and Methods

An STL file of an endodontic upper central incisor was extracted from the digital bank of the UCV, which was printed on a 3D printer and carved, leaving a minimum ferrule of 2mm, which was scanned to be able to perform group 1 and 2, where Group 1: 30 endocrowns were made, for Group 2: 30 crowns with core and post anatomically adapted to the shape of the canal were made, and for Group 3 they were cemented with a fiber post and stump reconstruction. These groups were scanned to design the final crown STL file. Subsequently, each group was subjected to compressive strength until fracture, in a ZwickRoell Z100 universal testing machine.

## Conclusions

The samples were tabulated and waiting for their statistics.

# Abstract: New impression-taking protocol for the manufacture of occlusal splints

**Authors:** C. Kunt

## Introduction

To establish a new impression-taking protocol for the manufacture of occlusal splints.

## Material and Methods

The patients who participated in the study were selected by the dentists working at the clinic of the Catholic University of Valencia. They are patients suffering from temporomandibular disorders and for their treatment an occlusal splint was recommended. The recordings required for the fabrication of the occlusal splints followed two different protocols and were made using the Primescan intraoral scanner (Dentsply Sirona) and the AFR mini REG occlusal device (Bausch) to determine the centric relation and adjust the vertical dimension. The principal investigator of the study was in charge of taking the records. The occlusal splints were designed and fabricated by the prosthodontist Isabel Cascales at the UCV clinic. On the day of delivery, the dentist responsible for the case filled in a quality survey which then allowed us to check whether a better quality unloading splint is obtained by means of the new recording protocol.

## Conclusions

The definitive results of the study are awaited.

# Abstract: Virtual Simulation for Skills Acquirement in the Cavity Carving

**Authors:** S. Arroyo Bote, T. Pereira, C. Bennasar Verger, S. Herrero Tarilonte, J. Mas Ramis, A.A. López González

## Introduction

The objective we are putting forward is to evaluate the usefulness of virtual reality in the acquirement of skills in the Cavitary Laboratory for conservative dentistry for students at dentistry level. Hypothesis (main objective): Virtual reality is an appropriate method for the teaching in dentistry of cavitary preparation. As secondary objectives, we shall analyse the following parameters: - Precision of preparation. - Surgery time and piercing methods employed. - Stages of preparation. - Volume and external objectives.

## Material and Methods

83 second, third and fourth-year students at the University College ADEMA (The University of The Balearic Islands), performed different practices in virtual simulators of perception Virteasy® in order to acquire the manual skills to control rotary instrumentation with specific dental tissue by employing the following methods: Second Course: Shaping dental tissues tracking letters E-D-C. Third Course: Type I cavity of Black in pits and fissures of the first inferior molar. Fourth Course: Type II cavity of Black of the proximal mesial face of a first inferior molar.

## Results

In agreement with the results of the ANOVA test, the differences observed in the accuracy of precision reported in the groups of the second, third and fourth courses are not significant (p-valor = 0.09915). On the contrary, what is significant is: the differences in surgery times observed among the separate groups: (p-valor=9.05910−7), Piercing times (p-valor=0.0001236), Progress (p-valor= 4.2610−8), Objective volumes (p-valor=1.2410−9) and external volumes (p-valor = 0.005844).

## Conclusions

All of the groups reached the objectives put forward with significant differences in surgery and piercing times, progress, objective volume and external volume. We conclude that virtual simulators of perception are a useful method in acquiring skills in the Cavitary Laboratory. 

# Abstract: Management of deep caries lesions

**Authors:** L. Domínguez Domínguez, I. Crespo Gallardo, O. Hay-Levytsk, J. Martín González, J.J. Segura- Egea

## Introduction

To determine the knowledge of Spanish dentists about diagnostic criteria and treatment decisions in the management of deep caries lesions (DCL) in daily clinical practice.

## Material and Methods

Spanish dentists were asked to respond to two surveys on diagnostic criteria and therapeutic decisions in the management of deep caries. The data were analyzed using descriptive statistics and the Shapiro-Wilk test, and odds ratios (OR) and confidence intervals (CI) were calculated as effect estimates.

## Results

Pulp sensitivity tests were used by 65% of dentists to assess pulp health in cases of DCL, particularly those who had followed cariology courses (OR = 3.8; p=0.005). Dentin hardness was the most frequently used criterion during DCL removal (98%). Two-thirds of respondents (65%) removed carious tissue completely with risk of pulp exposure (OR = 15.8; p=0.0000). Only 8% of dentists opted for partial caries removal, leaving some carious dentin near the pulp to avoid pulp exposure. More than half (51%) of the respondents considered that cariogenic microorganisms must be removed, or caries will progress, except for university dental professors (OR = 4.6; 95% CI = 1.3 - 15.8; p=0.017). Good clinical outcome was the most chosen reason (83%) for choosing a specific treatment.

## Conclusions

Spanish dentists are not using a conservative approach to the treatment of deep caries lesions in their daily clinical practice, and there is endodontic overtreatment. University professors are up to date in cariology, but it is necessary to propose new educational strategies on caries and its treatment so that practicing dentists can incorporate it into their daily clinical practice.
